# Ogilvie Syndrome in the Setting of Myxedema Ileus: A Case Report

**DOI:** 10.5811/cpcem.47381

**Published:** 2025-10-22

**Authors:** Sophia Mounce, Sharon H. Kim, James Waymack

**Affiliations:** * Southern Illinois University, School of Medicine, Springfield, Illinois; † Southern Illinois University, School of Medicine, Department of Emergency Medicine, Springfield, Illinois

**Keywords:** Ogilvie syndrome, myxedema ileus, hypothyroidism, case report

## Abstract

**Introduction:**

Ogilvie syndrome is described as the dilation of the colon without a clear mechanical obstruction. One predisposing factor to Ogilvie syndrome is hypothyroidism. The hypothyroid state can cause decreased gastrointestinal motility; however, hypothyroidism resulting in Ogilvie syndrome is a rare complication and is referred to as myxedema ileus. A review of literature shows limited reports of this specific process and none in the emergency medicine literature.

**Case Report:**

A 54-year-old woman with a history of hypothyroidism presented to the emergency department with three days of fatigue, generalized weakness, chills, diarrhea, and shortness of breath without chest pain or cough. Lab work showed high levels of ultra thyroid-stimulating hormone and decreased thyroid hormone levels. A computed tomography angiography of the chest, abdomen and pelvis showed multiple dilated loops of large bowel. Ultimately, she was diagnosed with pseudo-obstruction (Ogilvie syndrome) secondary to myxedema ileus.

**Conclusion:**

Ogilvie syndrome in the setting of myxedema ileus is a serious complication that may occur in patients who are in a severe hypothyroid state. It is important for emergency physicians to consider hypothyroidism as a potential cause of intestinal pseudo-obstruction.

## INTRODUCTION

Colonic pseudo-obstruction, also known as Ogilvie syndrome, is dilation of the colon without a clear mechanical obstruction that was first described in 1948 by Sir Heneage Ogilvie. The exact pathophysiology of this condition is not fully understood; however, it is thought to be due to imbalance in autonomic innervation.^1^ There are many potential causes and predisposing factors to Ogilvie syndrome. In a retrospective study of 400 patients with Ogilvie syndrome, the predisposing factors identified included infection, trauma, surgery, and miscellaneous medical conditions (metabolic, cancer, respiratory failure).^2^ Another potential metabolic predisposing factor includes hypothyroidism. Severe hypothyroidism can cause decreased gastrointestinal motility resulting in Ogilvie syndrome, which is a rare complication and is sometimes referred to as myxedema ileus.^3^ We report a case of Ogilvie syndrome secondary to myxedema ileus in a 54-year-old female with history of hypothyroidism. A review of the literature shows few case reports of this specific process and none in the emergency medicine literature.

## CASE REPORT

A 54-year-old woman with a history of hypothyroidism, diabetes mellitus type two, chronic obstructive pulmonary disease, asthma, anxiety, and depression presented to the emergency department (ED) via emergency medical services with three days of fatigue, generalized weakness, chills, and shortness of breath without chest pain or cough. Her symptoms had been progressively worsening, and she stated that she felt as if she could not move her body. She also noted diarrhea without abdominal pain, melena, or hematochezia. Prior to arrival, the patient’s daughter noted that the patient appeared paler and had dyspnea and a syncopal episode. The patient reported two missed doses of levothyroxine. On examination, her temperature was 36.4 °C, blood pressure 106/64 millimeters of mercury (mm Hg), heart rate 62 beats per minute, and respiratory rate 16 breaths per minute with oxygen saturation of 96% on room air.

On physical examination, the patient appeared drowsy. She was sitting with her eyes closed and was slow to answer questions, without any obvious distress. Her abdomen was soft, non-distended, and with normal bowel sounds. She noted diffuse abdominal discomfort to palpation; however, she stated this was chronic for her. She was oriented to person, place, time, and situation without any focal neurological deficits. Her strength was 3/5 throughout all extremities. The extremities were without edema.

Laboratory evaluation by point-of-care venous blood gas showed a pH of 7.28 (reference range: 7.35–7.45), partial pressure of carbon dioxide level of 60 mm Hg (41–51 mm Hg), bicarbonate level of 28.2 millimoles per liter (mmol/L) (24.0–28.0 mmol/L). Complete blood cell count and comprehensive metabolic profile were grossly unremarkable, and creatine kinase level was 333 international units per liter (IU/L) (30–223 IU/L). Thyroid studies showed an elevated thyroid-stimulating hormone (TSH) of 196.80 IU/mL (0.45–5.33 IU/mL). Free triiodothyronine (T3)/thyroxine (T4) and total T3/T4 were not resulted during the patient’s ED stay but were found to be significantly decreased upon admission: free T3 1.2 picograms per milliliter (pg/mL) (2.4–4.4 pg/mL), total T3 < 25 nanograms per deciliter (ng/dL) (87–178 ng/dL), free T4 0.2 ng/dL (refe0.5–1.3 ng/dL) and total T4 < 1.0 micrograms/dL (mcg/dL) (6.1–12.2 mcg/dL). Cortisol level was 1.7 mcg/dL (8.7–22.4 mcg/dL).

**Image 1:**
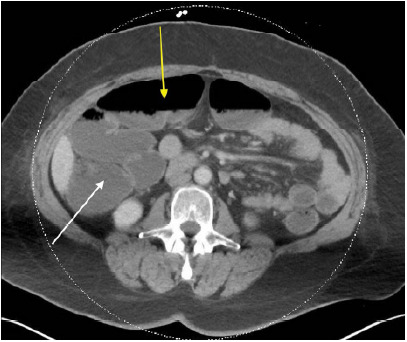
Computed tomography of the abdomen and pelvis (axial) demonstrating dilated large bowel/ascending colon (white arrow) with air-fluid levels (yellow arrow).

**Image 2:**
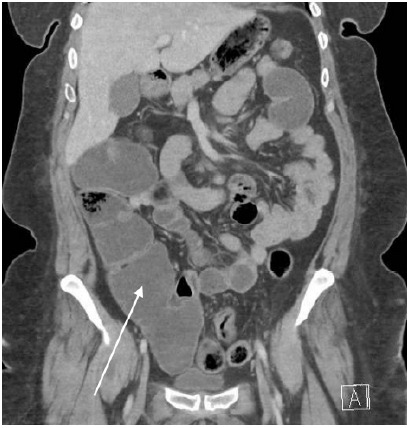
Computed tomography of the abdomen and pelvis (coronal) demonstrating dilated, fluid-filled large bowel/ascending colon (arrow) without obvious obstruction or transition point.

A computed tomography angiography (CTA) of the chest with routine abdomen and pelvis showed multiple dilated loops of large bowel and a few loops of distal small bowel with air-fluid levels (Images 1 and 2). Given this patient’s history, TSH level, and dilated bowel loops of large bowel on the CTA without a transition point, a diagnosis of pseudo-obstruction (Ogilvie syndrome) secondary to myxedema ileus was made. The few loops of dilated small bowel present were presumed to be sequelae of the large bowel dilatation.

*CPC-EM Capsule*

What do we already know about this clinical entity?

*Colonic pseudo-obstruction (Ogilvie syndrome) is colon dilation without mechanical obstruction, often after surgery, trauma, or severe illness*.

What makes this presentation of disease reportable?

*Hypothyroidism leading to Ogilvie syndrome is a rare complication referred to as myxedema ileus*.

What is the major learning point?

*Myxedema ileus should be suspected in patients presenting with Ogilvie syndrome and signs of severe hypothyroidism*.

How might this improve emergency medicine practice?

*Early recognition of myxedema ileus allows prompt initiation of thyroid hormone therapy, which can reverse colonic pseudo-obstruction and avoid complications*.

The patient remained stable in the ED and was admitted to the intensive care unit (ICU). Initial treatment in the ICU included 100 mg of intravenous (IV) hydrocortisone and 200 mcg of IV levothyroxine with 12.5 mcg of liothyronine. Nasogastric tube placement was deferred due to lack of vomiting, and the patient was started on a liquid diet while her hypothyroid condition was treated. The following day her symptoms and mental status improved, and she was downgraded to the intermediate care unit. At that time, she was started on a maintenance weight-based dose of IV levothyroxine. On day three of the patient’s hospitalization, her diet was advanced, and she was transitioned to oral medications. She was discharged one day later.

## DISCUSSION

Myxedema ileus due to hypothyroidism is a rare but serious cause of Ogilvie syndrome. There are few case reports in the literature.^4^^–8^ Classically, Ogilvie syndrome presents with marked constipation or obstipation. We believe the patient’s diarrhea was related to overflow in the setting of the acute colonic pseudo-obstruction as evidenced by CT findings of large-bowel dilatation without transition point or other signs of obstruction. While our patient stated the abdominal tenderness found on exam was chronic, it is likely her pain was related to her diagnosis of Ogilvie syndrome.

The current standard of care for myxedema ileus is conservative management with nasogastric tube decompression, bowel rest, and resumption or commencement of levothyroxine. Surgical intervention should be considered in cases with cecal distention of more than 12 cm, bowel ischemia, or perforation.^4^ While this is an uncommon complication, it should be considered as a differential diagnosis in patients with hypothyroidism who are presenting with symptoms consistent with an ileus. If myxedema ileus is left untreated, it can lead to more serious consequences such as abdominal compartment syndrome and bowel perforation.^4^,^7^ This case highlights the importance for emergency physicians to consider myxedema ileus as a potential diagnosis in patients with Ogilvie syndrome or a history of hypothyroidism.

## CONCLUSION

Myxedema ileus, pseudo-obstruction (Ogilvie syndrome), is a serious complication that may occur in patients who are in a hypothyroid state. It is important for emergency physicians to consider hypothyroidism as a potential cause of intestinal pseudo-obstruction. The treatment for these patients includes correction of the underlying endocrine pathophysiology and supportive care.
